# A novel biomarker selection method using multimodal neuroimaging data

**DOI:** 10.1371/journal.pone.0289401

**Published:** 2024-04-04

**Authors:** Yue Wang, Pei-Shan Yen, Olusola A. Ajilore, Dulal K. Bhaumik

**Affiliations:** 1 Division of Epidemiology and Biostatistics, University of Illinois at Chicago, Chicago, IL, United States of America; 2 Department of Psychiatry, University of Illinois at Chicago, Chicago, IL, United States of America; Chinese Academy of Medical Sciences and Peking Union Medical College, CHINA

## Abstract

Identifying biomarkers is essential to obtain the optimal therapeutic benefit while treating patients with late-life depression (LLD). We compare LLD patients with healthy controls (HC) using resting-state functional magnetic resonance and diffusion tensor imaging data to identify neuroimaging biomarkers that may be potentially associated with the underlying pathophysiology of LLD. We implement a Bayesian multimodal local false discovery rate approach for functional connectivity, borrowing strength from structural connectivity to identify disrupted functional connectivity of LLD compared to HC. In the Bayesian framework, we develop an algorithm to control the overall false discovery rate of our findings. We compare our findings with the literature and show that our approach can better detect some regions never discovered before for LLD patients. The Hub of our discovery related to various neurobehavioral disorders can be used to develop behavioral interventions to treat LLD patients who do not respond to antidepressants.

## Introduction

Patients with late-life depression (LLD) are usually over 55 years old and have major depressive symptoms. In the U.S., the prevalence of major depression in adults 50 years and older is estimated to be 4.7% [1]. As the world population of adults aged 60 years and older is expanding rapidly from 900 million in 2015 to more than 2 billion by 2050 [2], focus on understanding age-related disorders is becoming prominent and necessary to accommodate this demographic shift. One of the major disorders in elders is depression associated with socioeconomic, psychiatric, and medical factors [3]. Prominent depressive symptoms of LLD are severe, with persistent low mood and self-esteem, deep sadness, or a sense of despair. LLD patients lack interest in a previously rewarding or enjoyable activity. LLD is also a leading cause of disability in older adults and causes substantial health care expenses [4].

Detection of brain networks may serve as neuroimaging biomarker for disease identification and provide a better understanding of how to treat LLD patients. No specific guideline suggests how to identify LLD patients with corresponding severity early. The development of biomarkers utilizing brain network properties for early detection of LLD is needed to achieve optimal benefits while treating LLD patients. Identification of biomarkers and predictors becomes challenging due to the heterogeneity in brain tissues, choice of a subnetwork for group comparisons, and proper interpretation of performance function. The complexity of the current investigation is due to sample variability, the dependency between regions of interest (ROI), and high dimensionality for the non-Gaussian scenario or non-linear relationship in ROIs [5].

Diffusion tensor imaging (DTI) and resting-state functional magnetic resonance imaging (rs-fMRI) for the whole brain are used to measure structural connectivity (SC) and functional connectivity (FC), respectively. Abnormalities in FC and SC within the brain network involving cognitive function and mood regulation may increase the likelihood of developing depression. Currently, available approaches address one or, at most, a few of these statistical challenges, but none is able to address all comprehensively. The goal of this article is to identify functional biomarkers that may be potentially associated with the pathophysiology of LLD and address limitations of existing statistical methodologies frequently used to analyze such data.

Some studies used structural connectivity data to compare LLD with HC. DTI data is mainly used to measure structural connectivity. A study examined white matter structural alterations in specific brain regions implicated in depressed individuals compared to HC. A general linear model was proposed to identify SC changes in LLD patients. It found lower microstructural white matter activity in the right anterior cingulate cortex (ACC) and the right caudal middle frontal gyrus (also known as dlPFC) during episodes of depression in LLD patients [6]. Another study adopted a general linear model to observe the remission of depression. Results of that study showed higher fractional anisotropy (FA) in the frontal white matter above the anterior commissure-posterior commissure (AC-PC) plane of LLD individuals [7]. One study used a multiple linear regression model with tract-based spatial statistics to detect group differences and correlations of regional DTI data [8]. Another study used a hierarchical linear regression model to measure the association between white matter tracts and the ACC. A higher FA of left uncinate fasciculus was found in the LLD patients who suffered from apathy [9]. In addition, one study used a correlation analysis to confirm the association between SC and self-referential thinking [10].

However, the literature comparing LLD with HC using multimodal connectivity is very limited. One research proposed a linear mixed-effects model to detect biomarkers comparing LLD and HC by incorporating both measures of SC and FC and within-subject correlations. The study discovered disruptions in posterior and anterior parts [11]. Another study explored the relationship between brain connectivity and perceived loneliness in LLD patients. Using a general linear model combined with network-based Statistics (NBS), the findings of that study revealed that loneliness was associated with disrupted SC and FC between the amygdala and superior frontal gyrus [12]. The other research chose a Mann-Whitney U test to examine the group difference between LLD and HC or between LLD and remitted patients with LLD. Results of this study revealed that LLD patients had higher mean diffusivity in the white matter tract between the left ACC and posterior superior temporal gyrus (pSTG). Compared to the HC, the remitted patients with LLD had lower FC between the left ACC and pSTG; there is no significant difference between unremitted LLD and HC [13].

This article introduces a neuroimaging study to detect network alternations in LLD compared to HC. We develop a Bayesian mixture model for FC measures incorporating SC measures as auxiliary information. Our decision criteria for determining significant biomarkers is based on a proposed algorithm that utilizes information obtained from a Bayesian mixture model we developed. Further, it strictly controls the overall false discovery rate (FDR). We present results regarding disrupted FC of LLD compared to HC and hubs of disruptions with neurobehavioral activity. The results focus on the FC patterns within and between the major brain networks associated with LLD. Finally, we discuss some limitations of the current framework and provide suggestions and thoughts for future work.

## Materials and methods

### A multimodal neuroimaging study for late-life depression

A cross-sectional neuroimaging study was conducted by Dr. Ajilore at the University of Illinois at Chicago (UIC) to compare LLD with HC. For this study, a total of 42 participants were recruited. 21 participants in the LLD group were matched with another 21 participants in HC. All study participants were at least 55 years old.

### Participants of the study

The ages of participants ranged from 55 to 82 years, with a mean age of 67.8 years and a standard deviation of 7.05 years. There was a significant difference in age between the two groups (p = 0.001). These two groups had no significant difference regarding demographic characteristics, including sex, race, years of education, smoking status, and handedness. Additionally, there was no significant difference in clinical information, such as hemoglobin a1c, triglycerides, cholesterol, glucose, blood pressure, and stroke risk probability. These results suggest that HC and LLD groups were well-matched regarding demographic and clinical characteristics.

The recruitment method of participants were described in [14]. Individuals were recruited via community outreach (e.g., local newspaper, radio, and television advertisements) and relevant outpatient clinics. Initially, participants went through a screening procedure via telephone. The exclusion criteria were psychotic disorders (such as schizophrenia and bipolar disorder), history of any of the following: anxiety disorder outside of major depressive episodes, head trauma and substance abuse, and MRI contraindications (e.g., pacemaker, metal implants). The inclusion criteria for all participants were over 55 years of age, no history of unstable cardiac or neurological diseases, and medication-naive or anti-depressant free for at least two weeks. After the telephone screen, all eligible participants were scheduled for an assessment with Structured Clinical Interview for Diagnostic and Statistical Manual of Mental Disorders, 4^*th*^ edition (DSM-IV) (SCID-IV) [15] by a trained research assistant, followed by an evaluation on the severity of depression using the 17-item Hamilton Depression Rating Scale (HDRS) [16] by a board-certified psychiatrist. The LLD patients met SCID-IV criteria for major depressive disorder (MDD) and a score ≥ 15 on the 17-item HDRS. All study subjects provided written informed consent. The study was approved by the UIC Institutional Review Board and performed in compliance with the Declaration of Helsinki.

## Age effect on the identification of brain connectivity

Age has been identified as a potential confounding factor in studies investigating brain connectivity [8, 17]. Therefore, it is crucial to investigate the impact of age on identifying significant connectivity in this study. Using an FDR level of 0.30, 122, and 90 connectivities were identified by the hierarchical linear mixed effects model with and without controlling for age, respectively. Of these, 77 connectivity overlapped, resulting in an overlap rate of 85.6% (77/90). These findings suggest that the age effect does not have a huge impact on the identification of significant connectivity in the LLD study.

### Image acquisition and data processing

Brain regions in this neuroimaging study were parcellated by the Freesurfer Desikan atlas [18]. A total of 87 cortical with subcortical gray matter ROIs was considered for the whole brain analysis, including 43 bilateral regions and brain stem (central) in each participant. The FC and SC measures consist of a total of (872)=87×(87-1)/2=3741 unique links or connectivity.

For image acquisition, the neuroimaging data was processed by a Philips Achieva 3.0T scanner (Philips Medical Systems, The Netherlands) with an 8-channel sensitivity-encoding head coil. The participants were positioned on the scanner bed, fitted with soft ear plugs and foam pads, to ensure comfort and reduce head motion. During the scan, participants were required to remain still, keep their eyes closed, be relaxed, and not think anything particular. The three-dimensional T1-weighted image data was obtained with an MPRAGE (Magnetization Prepared Rapid Acquisition Gradient Echo) sequence. The parameters were as follows: relaxation time (TR) = 8.4 *ms*, excitation time (TE) = 3.9 *ms*, Field of View (FOV) = 240 *mm*, flip angle = 80°, voxel size = 1.1 × 1.1 × 1.1 *mm*. A single-shot gradient-echo echo-planar imaging (EPI) sequence was used to collect rs-fMRI imaging data with the following parameters: TR/TE = 2000/30 *ms*, FOV = 23 × 23 × 15 *cm*^3^, flip angle = 80°, EPI factor = 47, in-plane resolution = 3 × 3 *mm*^2^, slice thickness with no gap = 5 *mm*, slice number = 30, SENSE reduction factor = 1.8, NEX = 200; total scan time = 6:52. DTI imaging data were acquired using a single-shot spin-echo EPI sequence with the following parameters: TR/TE = 6994/71 *ms*, FOV = 240 *mm*, flip angel = 90°, voxel size = 0.83 × 0.83 × 2.2 *mm*. A total of 67 contiguous axial slices aligned to the anterior commissure-posterior commissure line was collected in 32 gradient directions with a *b* value of 700 *s*/*mm*^2^ and one acquisition without diffusion sensitization.

The artifact detection tool (ART: http://www.nitrc.org/projects/artifact_detect) was used to measure motion artifacts in all subjects. There was no significant between-group difference in composite motion (means the standard deviation(SD); HC: mean=.275, SD=.142, for LLD mean=.292, SD=.143, p=.77), still we controlled for any motion artifacts using realignment parameters detected by ART. There was no significant difference between HC and LLD subjects in movement. We used “scrubbing” to remove outlier frames as part of the CONN default processing pipeline.

The SC maps were generated for each participant using a pipeline integrating multiple image analysis techniques. The diffusion-weighted imaging (DWI) images were first corrected using the automatic image registration tool in DtiStudio software [19] by registering all DWI images to their corresponding b0 images with a 12-parameter affine transformation, followed by computation of diffusion tensors and deterministic tractography using fiber assignment by a continuous tracking algorithm. The label maps were generated using T1-weighted images with FreeSurfer.

For data processing, FC was measured using the rs-fMRI toolbox CONN. The CONN tool performed seed-based correlation analysis by computing the Pearson correlation coefficients between the BOLD time series measured the activation from a given ROI to all other ROIs. The BOLD signal was passed through a band-pass filter of 0.008 to 0.09 Hz. As the rs-fMRI connectivity data was measured using Pearson’s correlation coefficients, we applied Fisher’s *Z* transformation to stabilize the variance and approximate normal distribution for the transformed data [20–22]. We assumed a Poisson distribution on the DTI data measured by FA, essentially the estimate of fiber counts [11]. Next, the cube-root transformation was applied for normality.

### Statistical methods for data analysis

Constraint regression models (e.g., LASSO or elastic net regression) are frequently used to analyze neuroimaging data (e.g., fMRI, DTI, etc.) for selecting features or important biomarkers to explain disease-biomarker relationship [21]. Then, machine learning techniques are used to classify or predict the disease status of new subjects [5]. For group comparisons (e.g., LLD vs. HC), mixed-effects models together with multiple comparison is used for such data to control the false discovery rate of biomarkers [21]. In particular, fMRI data is analyzed by model-based methods such as cross-correlation analysis (CCA) [23–27] or statistical parametric mapping [28]), or data-driven methods such as independent component analysis (ICA) or principal component analysis [29–32]. Generally, SC is determined using DTI data, measured by FA, relative anisotropy, mean diffusivity, or anisotropic diffusion [33–39].

Mixed-effects models or graph theoretical approaches are used to analyze structural neuroimaging data for generating structural connectomes [40, 41]. Graph theoretical procedures provide additional flexibility to examine the global property of each subject’s network, which focuses on the subnetwork of nodes while examining local network properties [40]. For multimodal analysis, Bayesian approaches utilize SC as a prior for FC [42–45], and data-driven approaches such as joint ICA [46–54], partial least squares [55–58], or CCA [32, 59–62] are also used with or without prior information. An alternative is to use a bivariate mixed-effects model to jointly analyze fMRI and DTI data of LLD or Major Depressive Disorder (MDD) subjects addressing between-region correlations [11]. What is critically important is the implementation of an inferential procedure particularly suitable to control the false discovery rate while testing multiple networks generated from group comparisons (e.g., LLD vs. HC) [20].

Statistical methods for controlling the false discovery rate in neuroimaging studies, such as Efron’s Local false discovery rate (Lfdr) [63, 64] are developed using only one modality (e.g., FC), ignoring the potential influence of other modalities (e.g., SC, electroencephalogram, etc.) completely. For this LLD study, we develop an analytical approach consisting of two steps. In the first step, we utilize a linear mixed-effects regression model to analyze FC and SC data separately to determine between-region connectivity, addressing correlations over regions and heterogeneity between subjects. Using this model, we obtain test statistics for between-group comparison of FC and SC. In the second step, we fit a two-group Bayesian mixture model [63–66] utilizing the density of FC test statistics with auxiliary information from SC test statistics.

We formulate a Bayesian mixture model for two-group comparison assuming that for connectivity links under the null hypothesis (i.e., no FC difference between disease and control group), referring to as null connectivity links, the absolute value of *t*-statistic (denoted by $|t_{i}^{\left(F\right)}|$) follows a folded normal distribution (denoted by $f_{0}\left(\right|t_{i}^{\left(F\right)}\left|,0,\sigma^{2}\right)$ with zero mean and an unknown variance *σ*^2^. This folded normal under the null distribution does not use any information from the SC. Next, we assume that connectivity links under the alternative hypothesis, i.e., a difference of FC exists between the disease group and control group (referred to as alternative connectivity links), the corresponding *t* statistic follows a gamma distribution denoted by $f_{1}\left(\right|t_{i}^{\left(F\right)}\left|,\alpha (\right|t_{i}^{\left(S\right)}\left|),\beta \right)$. The justification for using a gamma distribution is as it provides flexibility to accommodate different shapes of the data points and fits better for right-skewed data in general. We assume the shape parameter ($\alpha (|t_{i}^{\left(S\right)}|)>0$) of the gamma distribution is a log-linear function of the absolute value of the $|t_{i}^{\left(S\right)}|$ statistic of the difference of SC of two groups with a normal prior distribution. The rate parameter (*β*) of the gamma distribution does not depend on SC. A dichotomous latent weight variable *w*_*i*_ is attached to the mixture model where its zero value (i.e., *w*_*i*_ = 0) is assigned to the null distribution, and one (i.e., *w*_*i*_ = 1) is assigned to the alternate distribution. The prior probability of *w*_*i*_ = 1 depends on the SC statistic, i.e. $|t_{i}^{\left(S\right)}|$ is modeled using a logistic regression with $|t_{i}^{\left(S\right)}|$ as a covariate. By Bayes theorem, the Bayesian local false discovery rate, i.e. BLfdr_*i*_ ($|t_{i}^{\left(F\right)}|$, $|t_{i}^{\left(S\right)}|$)) is defined as the posterior probability that the *i*th connectivity link is null conditioning on $|t_{i}^{\left(F\right)}|$ and $|t_{i}^{\left(S\right)}|$ statistics. Thus our approach to modeling FC borrows strength from SC and provides a better assurance on the true detection of biomarkers.

Finally, utilizing the Bayesian information, we compute the Bayesian local false discovery rate (Blfdr) for each connectivity to decide its significance given the observed FC and SC test statistics. A detailed discussion of this statistical approach, including the simulation study to demonstrate the performance of the method in terms of controlling the FDR, is provided in [67]. In summary, we have extended the covariate-modulated Lfdr method [65] to multimodal neuroimaging data and implemented a Bayesian multimodal Lfdr approach to integrate SC and FC statistics utilizing a Bayesian mixture model (expression of the BLfdr is given in S1 Appendix). This approach leverages the complementary SC statistic as auxiliary information to enhance the modeling of the distribution of FC statistics to identify differential FC between two groups.

## Results

Bayesian multimodal Lfdr method detected 21 functional links of the LLD group that are significantly different from the corresponding functional links of the HC group at the FDR level *q* = 0.2 as shown in Table 1, where *q* is the desired FDR level with considerations on how to determine *q* described in [20, 68]. Among the 21 links, 15 have significantly higher FC (i.e., hyperconnectivity), and 6 have significantly lower FC (i.e., hypoconnectivity). In contrast, using FC data, Efron’s Lfdr method solely detects 12 significant functional links at *q* = 0.2, among which 11 links overlap with those identified by the Bayesian multimodal Lfdr method.

**Table 1:** Twenty-one differential functional links and the corresponding test statistics values determined by Bayesian multimodal Lfdr method at FDR level of 0.2 for LLD neuroimaging study.

Region 1^a^	Region 2^a^	Test statistic^b^
Hyperconnectivity:		
R thalamus proper (RTP)	R caudal middle frontal gyrus (RCMF)	4.340 (E)
R pallidum (RP)	L inferior parietal cortex (LIP)	4.011 (E)
R pallidum (RP)	R caudal middle frontal gyrus (RCMF)	4.051 (E)
R accumbens area (RAA)	L isthmus cingulate cortex (LIC)	3.890 (E)
R ventral diencephalon (RVD)	R fusiform gyrus (RF)	4.132 (E)
L isthmus cingulate cortex (LIC)	R caudal middle frontal gyrus (RCMF)	4.583 (E)
L posterior cingulate cortex (LPC)	R rostral middle frontal gyrus (RRMF)	3.877 (E)
L posterior cingulate cortex (LPC)	R supramarginal gyrus (RS)	3.378
L rostral middle frontal gyrus (LRMF)	R caudal middle frontal gyrus (RCMF)	4.074 (E)
L superior parietal cortex (LSP)	R pars opercularis (RPO)	3.355
R caudal anterior cingulate cortex (RCAC)	R caudal middle frontal gyrus (RCMF)	3.855 (E)
R caudal middle frontal gyrus (RCMF)	R isthmus cingulate cortex (RIC)	3.733 (E)
R caudal middle frontal gyrus (RCMF)	R posterior cingulate cortex (RPC)	3.598
R pars triangularis (RPT)	R rostral anterior cingulate cortex (RRAC)	3.469
R posterior cingulate cortex (RPC)	R supramarginal gyrus (RS)	3.491
Hypoconnectivity:		
L thalamus proper (LTP)	L posterior cingulate cortex (LPC)	-3.701
L ventral diencephalon (LVD)	R caudal anterior cingulate cortex (RCAC)	-3.889
R caudate nucleus (RCau)	L cuneus cortex (LCun)	-3.245
L entorhinal cortex (LE)	L supramarginal gyrus (LS)	-3.314
L fusiform gyrus (LF)	L pars triangularis (LPT)	-3.864
L fusiform gyrus (LF)	L supramarginal gyrus (LS)	-4.332 (E)

^*a*^ L = left; R = right.

^*b*^ Based on between-group comparison in FC using a linear mixed-effects regression model assuming random subject effect and heteroscedastic errors at both group and link level, a positive value indicates hyperconnectivity, a negative value indicates hypoconnectivity. 11 FC links were also identified by Efron’s Lfdr method at *q* = 0.2 are indicated using (E).

**Fig 1:**
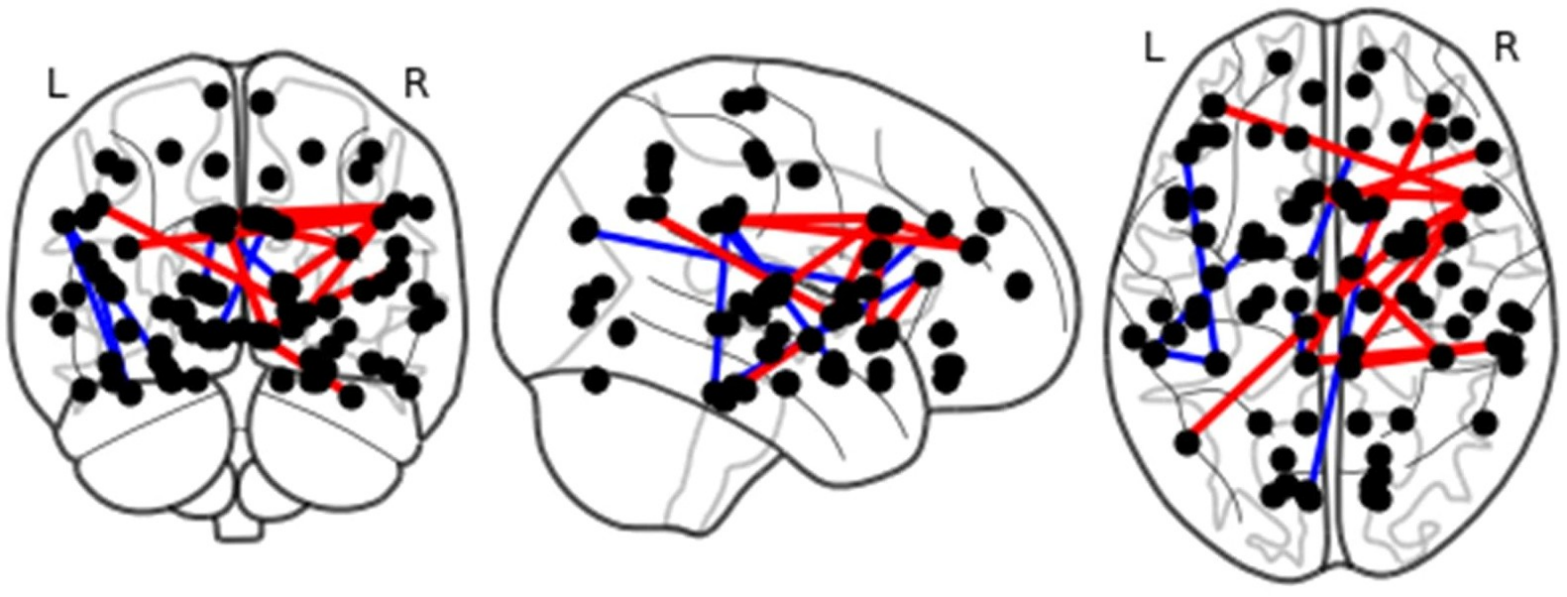
Brain images of 21 functional links exhibiting significant between-group differences identified by the Bayesian multimodal Lfdr method at an FDR level of 0.2. The red lines indicate higher FC (hyperconnectivity), and the blue lines indicate lower FC (hypoconnectivity). L = left; R = right.

**Fig 2:**
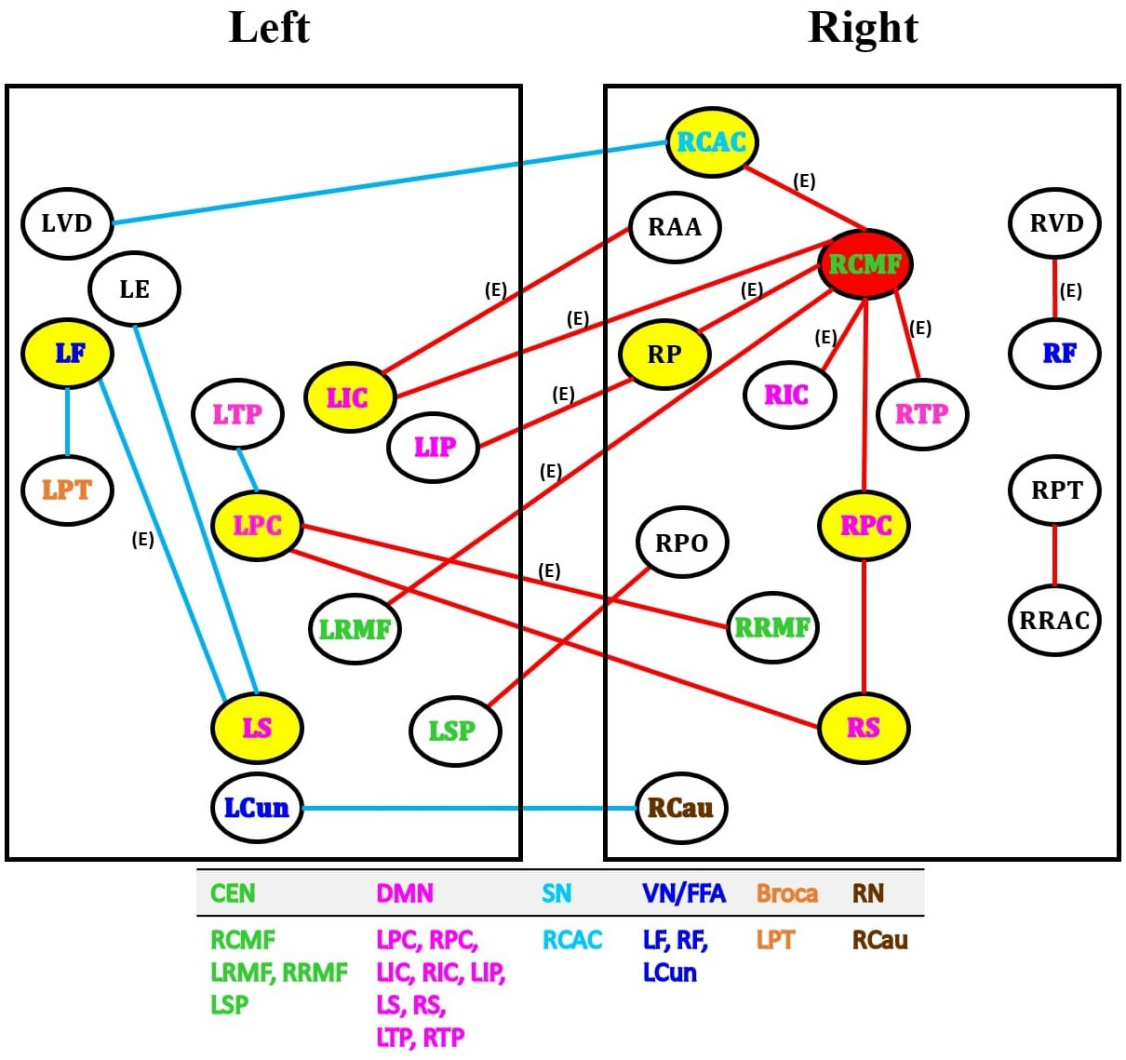
Network analysis of 21 functional links exhibiting significant between-group differences identified by the Bayesian multimodal Lfdr method at FDR level of 0.2 based on cortical and subcortical gray matter regions by the left and right hemispheres of the brain. Red lines indicate higher FC (hyperconnectivity), and the blue lines indicate lower FC (hypoconnectivity). The red circle denotes the primary hub, and the yellow circles denote secondary hubs. 11 FC links also identified by Efron’s Lfdr method are indicated using (E). Abbreviations: CEN = central executive network; DMN = default mode network; SN = salience network; VN/FFA = visual network / fusiform face area; Broca = Broca’s area; RN = reward network; LCun = left cuneus cortex; LE = left entorhinal cortex; LF/RF = left/right fusiform gyrus; LIC/RIC = left/right isthmus cingulate cortex; LIP = left inferior parietal cortex; LPC/RPC = left/right posterior cingulate cortex; LPT/RPT = left/right pars triangularis; LRMF/RRMF = left/right rostral middle frontal gyrus; LS/RS = left/right supramarginal gyrus; LSP = left superior parietal cortex; LTP/RTP = left/right thalamus proper; LVD/RVD = left/right ventral diencephalon; RAA = right accumbens area; RCAC = right caudal anterior cingulate cortex; RCMF = right caudal middle frontal gyrus; RCau = right caudate nucleus; RP = right pallidum; RPO = right pars opercularis; RRAC = right rostral anterior cingulate cortex.

Fig 1 presents the brain images of 21 functional links showing a significant difference between the LLD and HC groups by the Bayesian multimodal Lfdr method at *q* = 0.2. Fig 2 displays the network analysis of 21 functional links based on cortical and subcortical gray matter regions in the left and right hemispheres of the brain. The procedure of determining the significant link by the BLfdr is discussed in S1 Appendix.

First, we identify the primary hub region, the dlPFC, that has significantly higher FC to seven other regions, including left isthmus cingulate cortex, left rostral middle frontal gyrus, right thalamus proper, right pallidum cortex, right caudal anterior cingulate cortex, right isthmus cingulate cortex and right posterior cingulate cortex in LLD patients as compared to the HC group.

The second main finding is that LLD patients exhibit higher FC pattern comprising the right dlPFC, bilateral right rostral middle frontal gyrus within the CEN, right caudal anterior cingulate cortex within the SN, bilateral isthmus cingulate cortex, bilateral posterior cingulate cortex, right thalamus proper and right supramarginal gyrus within the DMN. We observed higher FC between the right supramarginal gyrus with the bilateral PCC region within the DMN. Lower FC was found between the left posterior cingulate cortex and left thalamus proper within the DMN. Also, we noticed higher FC between the left superior parietal cortex and right pars opercularis in LLD. We found lower FC between the left PCC and left thalamus proper within the DMN in the left hemisphere and higher FC in bilateral PCC regions. We also observed lower FC between the left pars triangularis, left FG, left supramarginal gyrus, and left entorhinal cortex in the LLD group, suggesting potential functional semantic and facial recognition inhibition in the left hemisphere of the brain. Our results show a consistently higher FC between the right dlPFC within the CEN, right dACC within the SN, and right PCC within the DMN in LLD patients.

In addition, we find eight secondary hubs, defined as regions showing at least two significantly higher or lower FCs to other regions. These regions are the left fusiform gyrus (FFA), right pallidum, right dACC (SN), left isthmus cingulate cortex (DMN), bilateral posterior cingulate cortex (DMN), and bilateral supramarginal gyrus (DMN). Moreover, significant FC activities are observed in eight bilateral regions: rostral middle frontal gyrus (CEN), dorsal thalamus, ventral diencephalon, fusiform gyrus (FFA), pars triangularis, isthmus cingulate cortex (DMN), posterior cingulate cortex (DMN) and supramarginal gyrus (DMN). We also observed lower FC between the right caudate nucleus and left cuneus cortex in the LLD group.

Inspecting Fig 2, we find that the hub of disruptions is the right dlPFC, which can be used as an intervention target for repetitive transcranial magnetic stimulation (rTMS). Future studies with larger samples should examine whether the right dlPFC would be a better intervention target for rTMS in LLD.

## Reproducibility of our results

To check the reproducibility of our findings, we used data from a neuroimaging study involving internalizing psychopathologies (IP) subjects. In that work, IP was characterized by disordered emotion processing, such as MDD, generalized anxiety disorder (GAD), and other related diagnoses. Study subjects were recruited from the greater Chicago area through UIC; the neuroimaging data processing procedure is described in [69]. To make a consistent comparison, we chose those IP patients who suffered from mild to severe depression symptoms. Specifically, 20 subjects with an HDRS score above 14 were chosen from the IP group. The propensity scoring matching method selected 23 subjects from the healthy control group. Study subjects aged were between 18 and 60 years, with a mean age of 27.4 years and a standard deviation of 11.0 years. The brain network was parcellated by CONN atlas [70], namely, to get a total of 105 brain regions with 5460 unique links. Comparing the IP study with the LLD study, we see that the age distributions of these two studies are different. To minimize the impact of age, subjects in each study were matched with ages for two comparison groups.

Efron’s Lfdr and Bayesian multimodal Lfdr detected respectively 45 and 3 (right amygdala—right middle temporal gyrus, right pallidum—left middle temporal gyrus, and right temporal fusiform cortex—left supramarginal gyrus) FC significant links while comparing the IP group with the HC group. The three links detected by the Bayesian method were also detected by Efron’s method. The links of IP and of LLD cannot be compared directly due to the difference in brain parcellation (i.e., 105 brain regions for IP vs. 86 brain regions for LLD). To bring into conformity, we compared those two groups’ brain regions (instead of links). Brain regions detected in both IP and LLD groups by the Bayesian Lfdr are the right pallidum and the left supramarginal gyrus. It is worth noting that the right pallidum is within the RN, and the left supramarginal gyrus is within DMN. Both RN and DMN have been detected in LLD. This brings a partial reproducibility result by the proposed Bayesian method.

## Model comparison with multimodality

Several investigators used multimodal neuroimaging data to better understand the neurological condition of brain with diseases. Honey et al. (2009) [71] investigated the correlation between HC (using resting state fMRI) with SC (using DTI). Sui et al. (2013) [72] employed the independent component analysis and estimated the number of independent components using three modalities such as fMRI, DTI, and structural MRI, while studying the abnormal structure underlying schizophrenia relative to healthy controls. Zhao (2014) [11] used a bivariate linear mixed-effects model (BLMM) to detect disrupted links of LLD compared to HC. Multimodality of neuroimaging studies mainly focuses on correlations of modalities or detection of the main components of such correlations.

For comparison, we developed a hierarchical bivariate linear mixed-effects model (HBLMM) extending Zhao’s model [11] where HC and FC were jointly used to detect disruptions as opposed to the proposed Bayesian model where SC is used as auxiliary information to model FC. The HBLMM detected forty links with significant group differences in HC and FC jointly. Two links, namely, the left ventral diencephalon (LVD)—right caudal anterior cingulate cortex (RCAC) and left thalamus proper (LTP)—left posterior cingulate cortex (LPC), overlapped with those detected by the Bayesian multimodal method. However, while considering the brain regions as a whole, there were 15 ROIs (LIP/LPC/RPC/LTP/RTP within the DMN, RCAC with the SN, LSP/RCMF/RRMF within the CEN, LCun/LF within the VN/FFA, and LVD/RAA/RCau/RP within the RN) detected by the HBLMM that overlapped with those found by the Bayesian multimodal method. The main difficulty with the HBLMM is the proper selection of the covariance matrix between HC and FC. Thus, findings may become subjective. Another difficulty of using HBLMM is the computational time due to the correlation matrix between HC and FC. Reviewing the literature, we find the proposed Bayesian approach is user-friendly and utilizes multimodality properly to detect functionally disrupted connectivity.

## Discussion

In this article, we have developed a rigorous analytical approach for detecting biomarkers using multimodal neuroimaging data. Our method uses SC information in defining the density function of the FC data under the alternative hypothesis that some FC of LLD differs significantly from the corresponding FC of HC. Both the null and alternative densities of FC are then used in the proposed Bayesian model to compute the Lfdr. As a result, adding strength from multimodal neuroimaging data, our Bayesian approach increases the sensitivity of testing the hypotheses that some FC of LLD differ from the corresponding FC of HC. It helps improve efficiency in controlling the FDR and detecting disrupted connectivity of LLD patients compared to HC patients. We find disrupted FC within and between some major brain networks, including CEN, DMN, SN, and fusiform face area, for facial cognition associated with LLD. CEN has been identified as a primary hub showing higher FC in CEN and DMN. These findings provide more detailed information on disrupted FC regions and patterns involving the underlying pathology of LLD and further insights on potential neuroimaging biomarkers for future clinical development to treat LLD patients. The robustness and reproducibility of our findings are verified via extensive simulation studies under the same parametric environment derived from the study data. It means that the chance is very low that we have detected false biomarkers. On the other hand, the likelihood of detecting the same biomarker is very high for detecting it with different LLD study data.

The dlPFC area has been implicated as the key neural substrate for MDD from the literature [73–75]. During working memory tasks, healthy subjects show bilateral activation in dlPFC and anterior cingulate cortex, while depressed patients exhibit asymmetric activity in dlPFC where the left dlPFC shows higher activation as reported in several neuroimaging studies [73, 76–78]. Alexopoulos et al. (2012) [79] found lower FC in the left dlPFC area in LLD patients relative to healthy subjects in an rs-fMRI study. The left dlPFC has been the target site of rTMS, a noninvasive procedure using magnetic field pulses to stimulate nerve cells, which was approved by the US Food and Drug Administration (FDA) in 2008 as a treatment for medication-resistant MDD (refer to FDA approval K061053). The clinical effectiveness of rTMS on the left dlPFC was established in randomized clinical trials for depression [80, 81]. By contrast, our analysis reveals evidence of the laterization of the right dlPFC with significantly higher FC and therefore suggests a specific and distinctive activation path via the right dlPFC in LLD patients. The right dlPFC was also a key hub of the altered FC in chronic neck pain patients at high risk of depression [82, 83]. Chronic pain is one of the most common co-occurring (i.e., comorbid) conditions in LLD patients [84]. Thus, the right dlPFC with higher FC is the key region involved in both cognition impairment and pain related to LLD.

Our findings align with the previous research with convergent data implicating disruptions of FC in the DMN in neural mechanisms for psychiatric disorders. A meta-analysis of rs-fMRI studies in MDD [85] reported higher FC within the DMN associated with MDD. A review of a number of rs-fMRI studies [86] showed reduced FC in the PCC region in healthy aging subjects. Also, higher FC has been reported in the posterior DMN area in major depression [87], and late-life anxious depression [88], in the PCC region within the DMN in LLD [79], in the left PCC region of DMN in MDD [89].

From the limited literature, we have learned that the disrupted right pars opercularisgyrus may be related to speech inhibition in a case reported [90], and hyperconnectivity of right pars opercularis was observed in adolescents with MDD [91]. Evidence shows that the right interior frontal gyrus, especially the right pars opercularis, may be specialized in music neurocognition [92]. It has long been known that music and emotion are connected, and music therapy has been applied to treat various psychiatric disorders such as depression [93].

Greicius et al. [94] have found higher FC in the thalamus with DMN during resting state in patients with MDD. Perrin et al. (2012) [78] had found that electroconvulsive therapy reduced FC between the right anterior cingulate cortex and right dlPFC in patients with severe depression. Yuen et al. (2014) [9] investigated the FC pattern of the SN in an rs-fMRI study to compare LLD with low and high apathy in healthy subjects, while apathy was a common symptom in MDD. Their results showed that relative to the HC group, LLD patients with high apathy had higher FC pattern within SN between the right anterior insular cortex and dACC and higher FC of the right anterior insular cortex to the right dlPFC within the CEN and right posterior cingulate cortex (BA 31) within the DMN.

Disrupted connectivity of bilateral FG to other regions may cause dysfunction in face recognition in patients with mild cognitive impairment. The right ventral diencephalon is important in predicting mild cognitive impairment and dementia in LLD patients [95]. In an fMRI study with healthy subjects [96], the left pars triangularis has been observed to activate in semantic processing. Further, another fMRI study of simultaneous language translation in healthy interpreters has reported higher activity in the left pars triangularis during backward translation. It suggests pars triangularis should be considered a “hub” of the language-control network [97]. A meta-analysis of previous studies on antidepressants [98] found that treatments had improved the neural response to positive emotion in the right dlPFC and left FG.

Our results show a higher FC pattern associated with LLD within and across the major large-scale neurocognitive brain networks, including CEN, DMN, and SN, which align with the previous findings of aberrant FC among large-scale brain networks in MDD [85]. In conclusion, our results suggest that LLD patients exhibit lower FC within the left hemisphere of the brain while higher FC is within the right hemisphere of the brain. This asymmetric FC pattern may be worth further investigation.

We envisage some limitations in the analytical approach that we have developed for biomarker detection. Our multimodal Bayesian approach controls the local false discovery rate for group comparisons using *cross sectional* neuroimaging data. As such, this approach cannot be used for longitudinal data. Over the past years, prospective longitudinal and interventional neuroimaging studies with patients having psychiatric or neurological disorders have gained popularity. For these studies, the primary objective is to investigate the association between changes in connectivity and those in clinical measurement (e.g., neurobehavioral measurement) within and between the intervention and control groups and evaluate the efficacy of the intervention. The multimodal neuroimaging and clinical data are collected at the baseline and scheduled post-baseline visits during the treatment period for each participant. Our analytical approach cannot be applied to such complex data to detect biomarkers.

We have the following suggestions for future work. We will need to substantially extend the current framework to handle more complex study designs and underlying data structures of prospective and interventional neuroimaging studies. The basic idea includes using a mixed-effects model for repeated measures to analyze longitudinal connectivity data and compare the intervention (e.g. LLD) and control groups. The subject-level demographics and baseline characteristics (e.g., age, baseline disease activity such as disease duration) can be included in the model as covariate(s). Test statistics for comparing LLD with HC are then modeled with other available modality data. One can compute Pearson correlation coefficients at each scheduled post-baseline visit to examine the association between connectivity and clinical measurements related to the intervention. These correlations can identify specific connectivity associated with clinical measures that are changing over time [99].

Machine learning and classification techniques are useful dimension reduction tools for high dimensional neuroimaging data involving thousands of connectivity collected from a limited number of subjects [100]. A predictive model based on variable selection methods can be applied to neuroimaging data to select connectivity that may differentiate the LLD group from the control group. Regularization approaches, such as LASSO [101], ridge [102], and elastic net regression [103], are intended to reduce variance at the cost of introducing some bias. The elastic net regression method may be a better choice for correlated variables, as it provides a compromise to balance between LASSO *l*_1_−norm penalty and ridge *l*_2_−norm penalty. Recent rs-fMRI neuroimaging studies have utilized elastic net logistic regression as a regularization method [21, 104]. Algamal and Lee [105] proposed an adaptive elastic net regularized logistic regression model in a high dimensional genomics study, which can also be applied to neuroimaging data. For LLD neuroimaging data with a small sample size of 23, splitting the already small data into even smaller training and testing data will be impractical. One solution is to develop data pooling strategies to combine the study data with other LLD neuroimaging studies using similar study designs [106] and then perform a meta-analysis of the pooled data using elastic net method [5].

In summary, using multimodal neuroimaging data, this article develops an analytical methodology and applies it to detect neuroimaging biomarkers for LLD patients. Our methodology is applicable for detecting biomarkers for other mental disorders such as anxiety, traumatic brain injury, and post-traumatic stress disorder. Biomarkers or hubs of disrupted FC provide insights into how the LLD group works differently than the HC group. Our findings are expected to help develop interventions to treat LLD patients better.

## Supporting information

S1 Appendix (PDF)

S1 Data (TXT)

S2 Data (TXT)
