# Supplementary material for: A novel biomarker selection method using multimodal neuroimaging data
Source: PLoS One. 2024 Apr 4;19(4):e0289401. doi: 10.1371/journal.pone.0289401 (PMC10994318; doi:10.1371/journal.pone.0289401)
Supplement: S1 Appendix — (PDF) [file pone.0289401.s001.pdf]

A Novel Biomarker Selection Method Using Multimodal Neuroimaging Data

Yue Wang[1], Pei-Shan Yen[1], Olusola A. Ajilore[2], Dulal K. Bhaumik[1,2*]

1 Division of Epidemiology and Biostatistics, University of Illinois at Chicago, Chicago, IL, USA
2 Department of Psychiatry, University of Illinois at Chicago, Chicago, IL, USA

* dbhaumik@uic.edu

Appendix

We define $t_i^{(F)}$ and $t_i^{(S)}$ as the t statistics for measuring the differences between the ith FC and SC, respectively, between patients with LLD and HC. Under the null hypothesis, the absolute value of $|t_i^{(F)}|$ follows a Folded Normal distribution with mean $\mu_0 = 0$ and variance σ^2, denoted by $f_0(|t_i^{(F)}|, 0, \sigma^2)$. Under the alternative hypothesis, the absolute value of $|t_i^{(F)}|$ follows a Gamma distribution with shape parameter $\alpha(|\ t_i^{(S)}\ |) = exp(\alpha_1 + \alpha_2 |t_i^{(S)}|) > 0$ and scale parameter β, denoted by $f_1(|t_i^{(F)}|, \alpha(|t_i^{(S)}|), \beta)$.

The prior probability of occurrence of the alternative hypothesis, $\pi_{i1}\big(|t_i^{(S)}| \;\big|\; \boldsymbol{\gamma}\big)$, is given by $P(\omega_i = 1 \;\big|\; |t_i^{(S)}|) = \frac{exp(\gamma_0+\gamma_1 |t_i^{(S)}|)}{1+exp(\gamma_0+\gamma_1 |t_i^{(S)}|)}$. For the other unknown parameters $\{\boldsymbol{\alpha}, \boldsymbol{\gamma}\}$, we assume generic weakly informative prior distributions providing more stable results compared to non-informative prior, but still have enough vagueness to ensure that the data dominate the posteriors. For $\boldsymbol{\alpha} = [\alpha_0 \quad \alpha_1]^T$ and $\boldsymbol{\gamma} = [\gamma_0 \quad \gamma_1]^T$, we assume that α_0 and α_1 are *a priori* independent, and also γ_0 and γ_1 are *a priori* independent, and their values are concentrated between -1 and 1. Further we assume $\boldsymbol{\alpha} \sim N_2\left(\mathbf{0}, \Sigma_{\boldsymbol{\alpha}}\right)$ and $\boldsymbol{\gamma} \sim N_2\left(\mathbf{0}, \Sigma_{\boldsymbol{\gamma}}\right)$, where $\Sigma_{\boldsymbol{\alpha}} = \Sigma_{\boldsymbol{\gamma}} = \begin{bmatrix} 1 & 0 \\ 0 & 1 \end{bmatrix}$. Based on the posterior estimates of the model parameters $\{\hat{\boldsymbol{\alpha}}, \hat{\boldsymbol{\gamma}}, \hat{\beta}, \hat{\sigma}_0^2\}$, we calculate the posterior probability of each individual link given the observed FC and SC test statistics, $\widehat{BLfdr}_i$, $i = 1, ..., 3741$, (expression is given below), and then determine which connectivity links to be rejected using the oracle procedure for FDR control [**?**]. We refer to [**?**] for a full description of the BLfdr procedure with each prior and posterior distributions.

The estimated Bayesian local false discovery rate $\widehat{BLfdri}$ can be expressed as

$$\frac{\pi 0\left(|t_i^{(S)}|\middle|\hat{\boldsymbol{\gamma}}\right) f_0\left(|t_i^{(F)}|\middle|\hat{\sigma^2}\right)}{\pi_0\left(|t_i^{(S)}|\middle|\hat{\boldsymbol{\gamma}}\right) f_0\left(|t_i^{(F)}|\middle|\hat{\sigma^2}\right) + \pi_1\left(|t_i^{(S)}|\middle|\hat{\boldsymbol{\gamma}}\right) f_1\left(|t_i^{(F)}|\middle|\hat{\boldsymbol{\alpha}}, \hat{\beta}, |t_i^{(S)}|\right)}.$$
